# Collaborative strategies for deploying artificial intelligence to complement physician diagnoses of acute respiratory distress syndrome

**DOI:** 10.1038/s41746-023-00797-9

**Published:** 2023-04-08

**Authors:** Negar Farzaneh, Sardar Ansari, Elizabeth Lee, Kevin R. Ward, Michael W. Sjoding

**Affiliations:** 1 The Max Harry Weil Institute for Critical Care Research & Innovation, University of Michigan, Ann Arbor, MI USA; 2 Department of Emergency Medicine, University of Michigan Medical School, Ann Arbor, MI USA; 3 Department of Radiology, University of Michigan, Ann Arbor, MI USA; 4 Department of Biomedical Engineering, University of Michigan, Ann Arbor, MI USA; 5 Department of Internal Medicine, Division of Pulmonary and Critical Care, University of Michigan Medical School, Ann Arbor, MI USA

**Keywords:** Diagnosis, Respiratory distress syndrome

## Abstract

There is a growing gap between studies describing the capabilities of artificial intelligence (AI) diagnostic systems using deep learning versus efforts to investigate how or when to integrate AI systems into a real-world clinical practice to support physicians and improve diagnosis. To address this gap, we investigate four potential strategies for AI model deployment and physician collaboration to determine their potential impact on diagnostic accuracy. As a case study, we examine an AI model trained to identify findings of the acute respiratory distress syndrome (ARDS) on chest X-ray images. While this model outperforms physicians at identifying findings of ARDS, there are several reasons why fully automated ARDS detection may not be optimal nor feasible in practice. Among several collaboration strategies tested, we find that if the AI model first reviews the chest X-ray and defers to a physician if it is uncertain, this strategy achieves a higher diagnostic accuracy (0.869, 95% CI 0.835–0.903) compared to a strategy where a physician reviews a chest X-ray first and defers to an AI model if uncertain (0.824, 95% CI 0.781–0.862), or strategies where the physician reviews the chest X-ray alone (0.808, 95% CI 0.767–0.85) or the AI model reviews the chest X-ray alone (0.847, 95% CI 0.806–0.887). If the AI model reviews a chest X-ray first, this allows the AI system to make decisions for up to 79% of cases, letting physicians focus on the most challenging subsets of chest X-rays.

## Introduction

Recent advances in artificial intelligence (AI) have led to the development of AI models with human-level performance in the diagnoses of many health conditions based on clinical waveforms and images^1–3^, including radiographic images^4^. However, because medical diagnostic decisions are often high-stakes, completely replacing human expertise with automated AI models alone is unlikely to be acceptable by clinicians and patients^5^. Instead, strategies where an AI model collaborates with, rather than fully replaces a physician, may be a reasonable alternative. When optimized, such collaborations can result in higher diagnostic accuracy than either physicians or AI systems alone^6^.

A limited body of literature exists investigating how automated AI systems can collaborate with physicians for diagnostic decision-making. Several studies have examined strategies where an AI model’s prediction is shown to a physician before the physician makes the final diagnostic decision^6–9^. However, there may be scenarios where alternative strategies that better leverage the unique strengths of AI models and physicians may be more optimal. Understanding both the strengths and blind spots of AI algorithms and physicians could provide insights as to how AI and clinicians could most effectively collaborate together and help guide decisions surrounding AI model deployment to ensure safe, efficient, and effective use of AI systems to improve diagnostic decision-making.

This work examines possible strategies for AI model deployment on chest X-rays in the context of identifying acute respiratory distress syndrome (ARDS). ARDS is a common critical illness syndrome^10^ that develops in over 200,000 people in the United States every year^11,12^ and has a mortality rate of 40%^13^ yet diagnosis is commonly delayed or missed. Several studies have demonstrated that this syndrome is under-recognized in clinical practice resulting in patients not receiving evidence-based therapies^13^. ARDS is currently diagnosed based on the Berlin ARDS definition with the presence of bilateral airspace opacities on chest X-rays being a key criterion for the diagnosis^14–16^. However, physicians have been shown to have low reliability identifying findings of ARDS on chest X-rays^17^. Therefore, an AI tool could be of value to support clinicians with the interpretation of chest X-rays for ARDS.

In this study, we examine the strengths and weaknesses of physicians and a previously published deep learning algorithm^18^ for interpreting chest X-rays for ARDS to gain insight into potential strategies for physicians and an AI model to collaborate in the diagnosis of ARDS. Then, we evaluate several strategies for model deployment and physician-AI collaboration, including: (1) physicians interpreting chest X-rays first and deferring to the AI model if uncertain, (2) AI model interpreting chest X-rays first and deferring to a physician if uncertain, and (3) AI model and physician interpreting chest X-rays separately and then averaging, or weighting, their interpretations. While we find that the weighted average of AI and physician diagnoses leads to the highest accuracy, this strategy would require physicians to review all chest X-rays. Having the AI model interpret a chest X-ray first and defer to the physician if it is uncertain has near-equivalent accuracy, while reducing the number of chest X-rays that required manual physician review to determine if ARDS is present by 79.2%.

## Result

### Study cohort

The University of Michigan Institutional Review Board (IRB) approved this study (HUM00180748) with a waiver of informed consent from patient subjects and study physicians. The test dataset included 414 chest X-rays from 115 adult patients (age >17 years) who were consecutively hospitalized at a single health center in 2017 and developed acute hypoxic respiratory failure, defined as having a PaO_2_/FiO_2_ < 300 while receiving invasive mechanical ventilation or non-invasive mechanical ventilation. Demographics and clinical characteristics of the patient cohort are summarized in Table 1. Twelve patients who developed ARDS had initial chest X-rays that were not consistent with ARDS, but developed ARDS on a subsequent chest X-ray. The median number of chest X-rays per patient was 3 (IQR 2–4.5). Chest X-rays were performed a median 2 (IQR 1–4) days after patients were admitted to the hospital.

**Table 1:** Demographics of study patients.

Characteristic	Without ARDS	With ARDS	*P*-value
Patient, *n*	85	30	
Chest X-ray, *n*	326	88	
Age (years), median [Q1, Q3]	63 [55,72]	59 [49.25, 67]	0.23^a^
BMI (kg/m^2^), median [Q1, Q3]	26.94 [23.06, 32.69]	27.05 [24.34, 33.70]	0.75^a^
Sex			0.52^b^
Female, *n*	32 (37.65%)	14 (46.67%)	
Male, *n*	53 (62.35%)	16 (53.33%)	
Race			0.52^b^
Caucasian, *n*	71 (83.53%)	27 (90%)	
Black, *n*	11 (12.94%)	3 (10%)	
Other or unknown, *n*	3 (3.53%)	0 (0%)	
Risk factor			
Pneumonia, *n*	14 (16%)	7 (23%)	0.57^b^
Sepsis, *n*	13 (15%)	8 (26%)	0.27^b^
Trauma, *n*	6 (7%)	0	0.31^b^
Aspiration, *n*	3 (4%)	4 (13%)	0.14^b^
Acute hypoxemic respiratory failure (AHRF) severity (PaO_2_/FiO_2_)			<0.01^b^
200–300, *n*	43 (51%)	6 (20%)	
100–200, *n*	29 (34%)	12 (40%)	
<100, *n*	13 (15%)	12 (40%)	
Respiratory support			0.58^b^
Mechanical ventilation, *n*	84	30	
BiPAP, *n*	1	0	

^a^Welch’s *t* test is used to test for a significant difference between the mean of with and without ARDS populations.

^b^Chi-squared is used to test for a significant difference between the frequencies of categories with and without ARDS populations. *Q1* first quartile, *Q3* third quartile, *BMI* body mass index, *ARDS* acute respiratory distress syndrome, *BiPAP* bilevel positive airway pressure.

### Comparing the AI model to physicians

Chest X-rays performed when patients met criteria for acute hypoxic respiratory failure were reviewed by six or more physicians with expertise in the identification of ARDS, while physicians also reviewed other relevant clinical information for each patient. Their combined reviews served as a reference standard for whether ARDS findings were present. This reference standard was used to evaluate the AI model performance and individual physicians. In total, 9 physicians reviewed chest X-rays for the study, each reviewing between 123 and 414 chest X-rays (see Method for details).

Overall, the deep learning-based AI system had significantly higher accuracy in detecting ARDS images compared to physicians (0.847 [95% CI 0.806–0.887] vs. 0.808 [95% CI 0.767–0.85], *p* value of < 0.05 using the one-sided bootstrapped two-sample hypothesis testing) (Table 2). Physicians had lower average sensitivity (0.727 [95% CI 0.651–0.796]) across the chest X-rays that they reviewed compared to the AI model’s sensitivity (0.789 [95% CI 0.679–0.89]) on the same chest X-rays. The AI model also maintained a higher specificity (0.864 [95% CI 0.816–0.91]) than physicians (0.838 [95% CI 0.804, 0.874]). Supplementary Fig. 1 shows examples of chest X-rays with and without ARDS findings both correctly and incorrectly classified by the AI model.

**Table 2:** Performance of the ARDS detection strategies.

	Physician	AI	AI-aided physician	Physician- aided AI	Average of physician & AI	Weighted average of physician & AI
Accuracy (95% CI)	0.808 (0.767, 0.85)	0.847 (0.806, 0.887)	0.824 (0.781, 0.862)	0.869 (0.835, 0.903)	0.86 (0.822, 0.894)	0.871 (0.836, 0.905)
F1 score (95% CI)	0.628 (0.511, 0.73)	0.69 (0.562, 0.793)	0.649 (0.533, 0.749)	0.726 (0.621, 0.815)	0.71 (0.594, 0.802)	0.729 (0.617, 0.819)
Sensitivity (95% CI)	0.727 (0.651, 0.796)	0.789 (0.679, 0.89)	0.735 (0.664, 0.8)	0.796 (0.707, 0.876)	0.774 (0.689, 0.852)	0.795 (0.701, 0.879)
Specificity (95% CI)	0.838 (0.804, 0.874)	0.864 (0.816, 0.91)	0.856 (0.821, 0.89)	0.893 (0.855, 0.926)	0.89 (0.86, 0.92)	0.897 (0.863, 0.929)
PPV (95% CI)	0.676 (0.579, 0.768)	0.624 (0.471, 0.758)	0.684 (0.576, 0.779)	0.696 (0.562, 0.808)	0.724 (0.62, 0.818)	0.709 (0.579, 0.812)
NPV (95% CI)	0.92 (0.885, 0.949)	0.933 (0.893, 0.966)	0.922 (0.889, 0.95)	0.936 (0.904, 0.964)	0.932 (0.9, 0.958)	0.937 (0.904, 0.965)
Review burden (95% CI)	100 (100, 100)	0 (0, 0)	100 (100, 100)	20.7 (15.8, 25.8)	100 (100, 100)	100 (100, 100)

95% confidence intervals were generated using cluster-bootstrapping. Review burden is defined as the percentage of chest X-rays that need to be reviewed by physicians. *AI* artificial intelligence model, *CI* confidence interval, *PPV* positive predictive value, *NPV* negative predictive value.

While the AI model had higher overall accuracy than physicians, we hypothesized there may be chest X-rays where the AI model still had lower performance. For example, the AI model may more consistently and correctly classify chest X-rays that are easier to diagnose, but physicians may be better at recognizing subtle findings that are needed to classify more challenging chest X-rays. Understanding physician and AI system strengths, weaknesses, and blind spots could better inform optimal system deployment. We explored this concept in two ways, by comparing AI and physician accuracy after dividing chest X-rays into difficult and non-difficult categories, and after stratifying chest X-rays by whether physicians or the AI model had higher uncertainty.

**Fig. 1:**
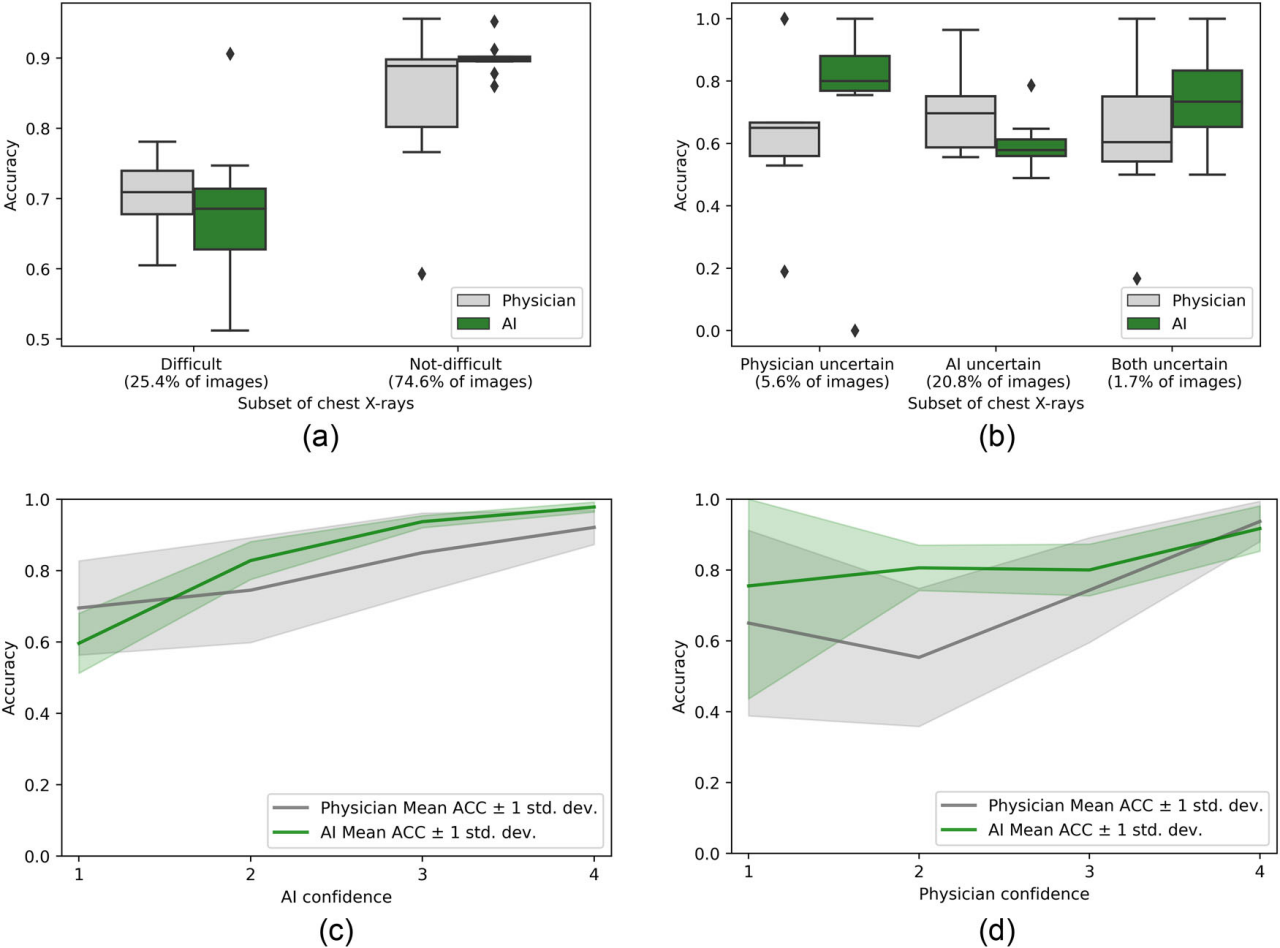
Performance of physicians and the AI model stratified on difficulty and certainty levels. **a** Compares the performance of physicians and AI performance stratified on the difficulty of the image interpretation, **b** compares the performance of physicians and AI stratified on uncertainty in their evaluation, and **c**, **d** show physician and AI accuracy stratified on their respective confidence levels. The physician accuracies were calculated by comparing each of the nine physicians against reference labels that were generated from the remaining eight physicians. Similarly, the AI accuracies were composed of nine values by comparing the AI model output against the same nine sets of reference labels that physicians were tested against. Bootstrapping was not used to generate Fig. 1. ACC accuracy, std. dev. standard deviation.

We identified difficult chest X-rays as those where at least two physicians disagreed with the majority label (after excluding the testing physician, see Method for details). In Fig. 1a, we plotted the variation in accuracy across physician reviewers, showing that physicians slightly outperformed the AI model in classifying the 25% of chest X-rays considered to be difficult by this definition. On average, physicians had an accuracy of 0.702 [95% CI 0.606–0.802] on these chest X-rays compared to an accuracy of 0.678 [95% CI 0.6–0.754] for the AI model. However, the AI system was notably more accurate in classifying the 75% of chest X-rays that were not difficult to classify (accuracy of 0.899 [95% CI 0.858–0.937] for AI vs. 0.844 [95% CI 0.807–0.882] for physicians), with less variability in accuracy compared to the group of physicians.

During their reviews, physicians graded each chest X-ray on a scale of 1–8, where 1 corresponded to no ARDS with high confidence, and 8 corresponded to ARDS with high confidence. Thus, a score of 4 or 5 indicated significant uncertainty in whether ARDS findings were present. Analogously, the AI model’s probability estimate range from 0.357 to 0.643, equivalent to a 4 or 5 on a 1–8 scale (see Method for details), could indicate model uncertainty. In Fig. 1b, we plotted variations in the accuracy of physicians and the AI model for chest X-rays with high and low certainty. Physicians indicated high uncertainty in only 5.6% of the chest X-rays on average, but in those groups of chest X-rays, the AI model was more accurate. On average, accuracy was 0.794 [95% CI 0.694–0.917] for AI vs. 0.626 [95% CI 0.5–0.744] for physicians. Similarly, physicians had higher accuracy in the larger subset of 20.8% of images in which the AI was uncertain (0.698 [95% CI 0.623–0.767] for physicians vs. 0.596 [95% CI 0.498–0.695] for AI) (Fig. 1b). There was also only a minimal number of chest X-rays (1.7%), where both AI and physicians were uncertain. These results provide evidence that AI and physician expertise can complement each other.

We also measured physician and AI accuracy as a function of their confidence levels (Fig. 1c, d). The AI model’s accuracy increased as its confidence increased, suggesting that the AI model is capable of recognizing when it is uncertain (green line in Fig. 1c). However, physicians demonstrated a lower ability to recognize when they should be uncertain, as seen by the gray line in Fig. 1d. Interestingly, this finding seemed to be driven by only a few physicians whose confidence levels were not well-calibrated to their accuracy (Supplementary Fig. 2). As shown in Fig. 1d, on average, the AI model outperforms physicians whenever physicians were not highly confident in whether a chest X-ray was consistent with ARDS (confidence level below 4). This result indicates that replacing physician diagnoses with AI when physicians are not highly confident (confidence level = 1, 2, or 3) resulted in greater accuracy than physicians alone.

### AI and physician collaboration

**Fig. 2:**
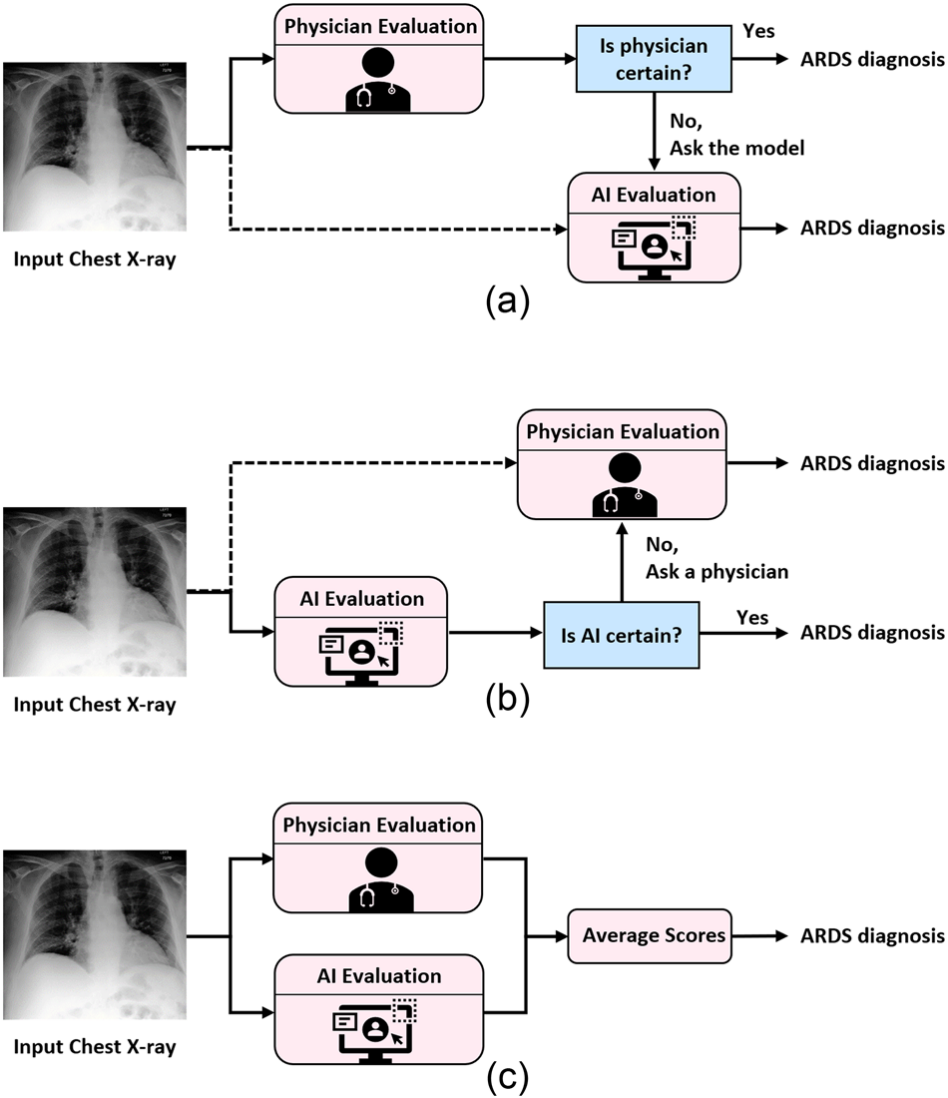
Schematic diagram of the proposed strategies for physician-AI collaboration. **a** Shows the proposed AI-aided physician framework, **b** shows the physician-aided AI framework, and **c** shows the average scoring framework. It can be both basic and weighted average.

Given the relative strengths and weaknesses of both physicians and the AI model for identifying findings of ARDS, we compared several collaborative strategies for combining physician and AI diagnostic expertise to understand which approach might result in the highest overall performance (Fig. 2). The strategies were:

1) *AI-aided physician:* physicians provide all the diagnoses which were replaced with the AI output score only if a physician was uncertain (Fig. 2a).

2) *Physician-aided AI:* AI was the primary diagnosis tool, and its diagnoses were replaced with physician reads only if AI was uncertain (Fig. 2b).

3) *Average of physician and AI:* this approach used the average ratings of both AI and physicians on a scale of 1 to 8 (Fig. 2c).

4) *Weighted average of physician and AI:* the weights were determined by maximizing the average validation accuracy (Fig. 2c).

**Fig. 3:**
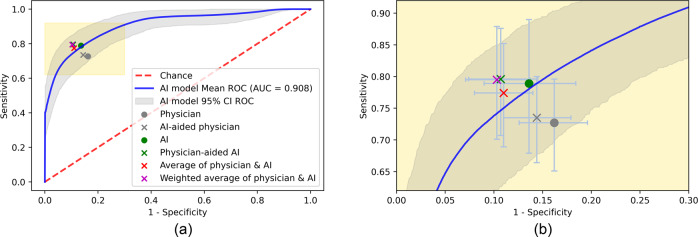
Average performance of physicians, AI, and four collaborative strategies. Receiver operating characteristic (ROC) curve of our AI model versus the performance of other strategies in ARDS detection. Markers denote each strategy’s performance in terms of sensitivity and 1-specificity along with their 95% CIs.

The two best performing strategies for maximizing diagnostic accuracy across all physicians evaluated were the physician-aided AI strategy (accuracy of 0.869 [95% CI 0.835–0.903]) and the strategy of taking a weighted average of the physician and AI model (accuracy of 0.871 [95% CI 0.836–0.905]) (Table 2 and Fig. 3). The weighted average of physician and AI model only marginally exceeded that of the physician-aided AI strategy, and would require physicians to review every chest X-ray. In contrast, in the physician-aided AI strategy, physicians would need to review only 20.8% of chest X-rays on average, thus off-loading the human expert workload on the reading of up to 79.2% chest X-rays, allowing physicians to focus on the more challenging subset.

Inspired by Oxipit ChestEye (the first AI-based framework for interpreting chest X-rays that was European Conformity (CE)-marked)^19–21^, we also evaluated an alternative strategy using an AI model as an auditing tool to identify potentially problematic physician reviews, which are then over-read by a second physician. In this framework, when the physician and AI disagreed, a second physician reviews the chest X-ray and the second physician’s review is used. This strategy did not result in meaningfully higher accuracy than the physician-aided AI approach (0.871 [95% CI 0.832, 0.909] vs. 0.869 [95% CI 0.835–0.903]). The auditing strategy requires all chest X-rays to be reviewed by a physician and an average of 22.9% reviewed by a second physician leading to a 122.9% review burden, which is much more physician resource intensive than the physician-aided AI approach which requires physicians to review only a subset 20.8% of chest X-rays.

### Physician-level analysis of collaboration strategies

**Fig. 4:**
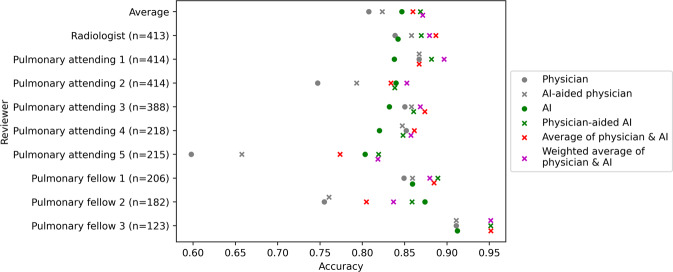
The accuracy of individual physicians, along with the performance of AI and four combinatory strategies when assessed on the same subset as physicians. Numbers in parentheses indicate the number of chest X-rays that each physician read.

We reviewed the collaboration strategies for each physician individually (Fig. 4, Supplementary Tables 1–3, and Supplementary Fig. 3). For 8/9 physicians the physician-aided AI strategy outperformed the physician’s alone accuracy. This physician-aided AI strategy was statistically significantly better than the physician’s accuracy alone for 3/9 physicians (Fig. 4, and Supplementary Table 1). Supplementary Fig. 3, and Supplementary Tables 2 and 3 also show the individual-level sensitivity and specificity values. The sensitivity of 3/9 physicians significantly improved after employing the physicians-aided AI strategy while this strategy did not result in a significant sensitivity decline for any physician, which means that the physician-aided AI strategy could improve rates of missed diagnosis.

## Discussion

This study examined the strengths and weaknesses of physicians and a previously published AI model^18^ for interpreting chest X-rays for ARDS. It also evaluated potential strategies for physician and AI model collaborations for ARDS diagnosis. We found evidence that AI and physician expertise can complement each other. When physicians were not confident in a chest X-ray’s interpretation, the AI model was often more accurate. In cases where the AI model had lower confidence, physicians were more accurate. The AI model had higher and more consistent accuracy on less difficult chest X-rays, while physicians had higher accuracy on difficult chest X-rays. The model was also generally better at calibrating its uncertainty, identifying a larger group of chest X-rays in which it was uncertain. These findings provide insight as to why collaborative strategies may improve rates of misdiagnosis. A strategy where the AI model reviews chest X-rays first and defers to clinicians when uncertain exceeded the physician- and AI-alone performance.

Several recent studies have demonstrated that deep learning-based AI systems can augment clinical decision-making. Providing deep learning model predictions to human experts improved medical image interpretation in different applications, including chest X-rays^7,8^, knee Magnetic Resonance Imaging (MRI)^9^, and skin cancer images^6^. However, all these studies relied on a physician to make a final decision after reviewing the model predictions. In contrast, our study explored several novel strategies for integrating physicians and AI by taking each method’s strengths and blind spots into account.

There are several ways that the collaborative strategies evaluated in this study could be deployed in practice for ARDS care. Simply making the AI model available to clinicians most closely aligns with the strategy of having clinicians review the chest X-ray first and then deferring to the AI model if uncertain. Our analysis suggests, however, that many times clinicians would likely feel confident in their evaluation of a chest X-ray and not consider or defer to the AI model for the assessment of ARDS. This approach potentially fails to leverage the AI model’s strengths to improve ARDS diagnosis and treatment at the bedside.

Many intensive care unit practices are highly protocolized by bedside nurses and respiratory therapists. Integrating the AI model into these protocols could be an effective way of deploying the model in practice. For example, one of the most important evidence-based practices in ARDS care is providing patients with lung-protective mechanical ventilation that uses tidal volumes based on ideal body weight. For patients with imaging findings consistent with ARDS, the system could set as a default low tidal volume setting and monitor patients to ensure they receive this therapy. It could communicate alerts to the respiratory therapist or nurses without significant physician oversight, only deferring to the physician in situations where the AI model has high uncertainty. This may be particularly helpful in low-resource settings, such as Intensive Care Units (ICU) without 24-hour access to critical care trained physicians.

The AI model could also be integrated into patient critical care dashboards. Dashboards are useful to clinicians, because they provide a quick, higher-level summary of critical information needed for patient care activities. AI models could abstract away the chest X-ray data, providing only the critical information in the dashboard (e.g., consistent with ARDS, inconsistent with ARDS, uncertain), allowing the clinician to quickly make use of this information without the need to review each chest X-ray for those details.

Our study has several limitations. First, this study was performed retrospectively and in a hypothetical manner based on previously collected chest X-ray review data. We did not evaluate a physician’s accuracy after directly showing them the AI prediction to allow them to synthesize this information in their evaluations. If physicians were told they could defer to the AI model if uncertain, they may have modified how they rated their certainty of diagnosis. The overall patient sample size and the number of ARDS cases were relatively low, potentially limiting generalizability. However, ARDS risk factors in this study were consistent with other ARDS epidemiology studies^13^. Due to the limited number of ARDS chest X-rays across patient demographic subgroups, we could not provide conclusive evidence for either accepting or rejecting the generalizability of the collaborative strategies across patient subgroups. When possible, transparent reporting on different subgroups is necessary in order to detect and avoid automation bias and protect underrepresented groups from worse-case scenarios and should be the subject of future work^22^. Finally, although we demonstrated evidence of the superiority of AI-based decisions, more research is still needed to investigate the collaboration of AI and physicians in other diagnosis tasks and environments.

While we aim to propose the most accurate and resource-efficient AI-physician collaboration strategy, we acknowledge that in high-stake clinical applications, our proposed physician-aided AI strategy is subject to stringent US Food and Drug Administration (FDA) regulatory requirements before it can be adopted in a real-world setting. Compared to AI systems that act as a recommendation system to physicians, the physician-aided AI strategy poses a higher level of autonomy by replacing physicians in the evaluation of chest X-rays for ARDS without supervision. Thus, this autonomy can be considered a barrier to its introduction into the practice. Physicians, however, will often still look at chest X-rays for more than just diagnosing ARDS, e.g., whether the endotracheal tube is in the right place or the presence of a pneumothorax. The ARDS model is not designed to assist in such activities, which could be considered another limitation of its use in practice.

In conclusion, this work suggests the potential value of integrating AI into clinical practice and using collaborative strategies between physicians and AI models to improve the diagnosis of ARDS. The physician-aided AI strategy, which defers the diagnosis to physicians only if the AI model is uncertain, resulted in improved diagnostic accuracy than both physician- and AI-alone. Automating recognition of ARDS for many patients may enable them to receive more consistent ARDS care.

## Method

### Patient cohort and data labeling

The 414 chest X-rays included in the test set were from 115 patients consecutively hospitalized between August 15 to October 2, 2017 at the University of Michigan who met criteria for acute hypoxemic respiratory failure (AHRF) in one of four intensive care units (medical, surgical, cardiac, and trauma). AHRF was defined as patients who had a PaO2/FiO2 < 300 while receiving invasive mechanical ventilation or non-invasive mechanical ventilation. A cohort flow diagram of the training, validation and test sets are shown in supplementary Fig. 4.

**Fig. 5:**
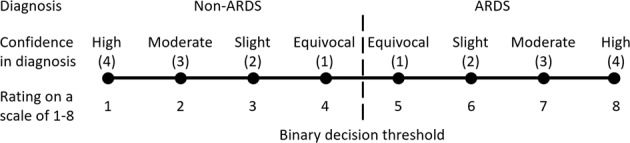
The ARDS assessment scale. A score of 1 indicates an absolute certain assessment as a non-ARDS chest X-ray, and 8 signifies an absolute certain ARDS chest X-ray. The binary decision threshold between non- ARDS, and ARDS is 4.5.

Frontal chest X-rays of patients in the test set were reviewed individually by at least six physicians for the (1) the presence or absence of ARDS and (2) confidence in this diagnosis while physicians also reviewed other relevant clinical information for each patient. Chest X-rays from the first 7 days after-admission were reviewed. Physicians were randomly assigned patients to review in the test set. Chest x-ray assessments were then converted to a rating on a scale of 1–8 as shown in Fig. 5. Six or more physicians reviewed each chest X-ray, and the majority voting was used to determine whether the chest X-ray was consistent with ARDS. In the event of ties, we averaged the ratings on a scale of 1–8, and any chest X-ray with an average value of ≥4.5 (binary decision threshold) was identified as consistent with ARDS. Nine physicians reviewed at least 100 chest X-rays including a radiologist, 5 pulmonary-critical care attendings, and 3 pulmonary-critical care fellows.

The overall reference labels were determined using all readings of an image and were used to generate Table 1. However, using the overall reference labels to assess a physician’s readings can artificially boost their performance since the testing physician can influence the ground truths against which they are tested. Consequently, when each physician was evaluated for their individual accuracy and accuracy of AI collaboration strategies, the reference labels were determined separately following the above-mentioned process but after excluding the test physician’s readings from the label set.

For each testing physician, a chest X-ray would be labeled as being uncertain if the physician states equivocal confidence in the diagnosis (confidence = 1, Fig. 5). Difficult chest X-rays were identified as those for which at least two physicians disagreed with the ground truth label. Note that, similar to deriving the ground truths, the testing physician is excluded when identifying disagreements.

### Deep learning model

We used a previously published deep convolutional neural network model with a 121-layer dense neural network architecture (DenseNet) to detect ARDS on chest X-rays^18^ with some modifications to the chest x-ray pre-processing and model pre-training steps. The chest x-ray pre-processing step was modified such that the image’s aspect ratio was maintained when re-scaling a raw image to a fixed-sized 320 by 320-pixel image. Pre-training on the CheXpert and MIMIC-CXR datasets was also increased to 15 epochs. The network weights were then retrained to detect ARDS following the published approach using the same data splits as in ref. ^18^ (Supplementary Fig. 4), where training and validation were performed on chest x-rays from a consecutive group of patients hospitalized with AHRF between January 2016 and June 2017 that were distinct from the hold-out set. There was no overlap between patients in the training/validation set and the 115 patients in the test set. After training, the AI model was calibrated using Platt scaling with data from the validation dataset, following the same approach as in its original development^23,24^.To align with the physicians’ rating scale, the model output probability scores were linearly mapped from 0–1 to a scale of 1–8. For this deep learning-based AI model, the uncertain images were identified as those with output scores of ≥3.5 and <5.5).

### Strategies for physician-AI collaboration

The four proposed frameworks for combining the physicians and AI diagnoses for ARDS detection are outlined in Fig. 2. These strategies are explained below in order from the least to the most level of automation involved.

1) In the *AI-aided physician* strategy (Fig. 2a), all chest X-rays would be reviewed by a physician first. However, if the physician was uncertain about a diagnosis (i.e., confidence = 1), the decision would be deferred to the AI model.

2) In the *Average of physician and AI* strategy (Fig. 2c), both physicians and AI would be asked to provide a rating on a scale of 1–8, which, as stated above, includes information about both the presence or absence of ARDS and the confidence level. The average of two scores would be used to make the final decision. In this strategy, the most confident of physician or AI will derive the decision.

3) The *Weighted average of physician and AI* strategy is similar to the simple averaging stated above. However, instead of using an equal weight, each of the physician and AI ratings were multiplied by a factor, *w*, reflecting its importance by

1 $$ARDS\,score = w_{{{{\mathrm{physician}}}}} y_{{{{\mathrm{physician}}}}} + w_{{{{\mathrm{AI}}}}} y_{{{{\mathrm{AI}}}}}$$

where *w*_AI_ = *1* *−* *w*_physician_*. y*_physician_ and *y*_AI_ correspond to the physician and AI ratings, respectively. For each physician, *w*_physician_ was estimated as the *weight* ∈ {0,0.05, 0.1,…, 1} that resulted in the highest average accuracy for the remaining eight physicians after excluding the testing physician. Except for pulmonary fellow 2, for all other eight physicians, *w*_physician_ was determined to be 0.3 (*w*_AI_ = 0.7). For pulmonary fellow 2, *w*_physician_ was 0.4 (*w*_AI_ = 0.6). The overall lower weight of physicians compared to AI indicates the greater importance of AI ratings. See Supplementary Fig. 5 for the detailed performance of individual and average physicians against different values of *w*_physician._

4) The AI model primarily provided the diagnoses in the *Physician-aided AI* strategy (Fig. 2b). Only in an event where AI is uncertain (output score ∈ [3.5, 5.5)) would a physician be making the diagnosis. This approach poses the highest level of automation (among four proposed strategies) by removing physicians from the loop in the evaluation of ~79.2% of chest X-rays.

We also evaluated a fifth combinatory approach proposed by Oxipit ChestEye^19–21^, where both physicians and the AI model evaluate a chest X-ray for ARDS findings. In the event that the AI model and physician disagreed, a second physician’s label is used. The second physician was randomly selected among all other reviewers reviewing the chest X-ray.

### Statistical analysis

We assessed the performance in terms of accuracy, F1 score, sensitivity, specificity, positive predictive value (PPV), and negative predictive value (NPV) after applying the binary decision threshold of 0.5 to the class probabilities or 4.5 (middle value) to the ARDS labels that range from 1 to 8. AUROC (area under the reciever operating chracteristic curve) is not used in this study since it would not be fair to compare the AUROC between physicians and the AI model. Physicians generate a binary score, and this score could not be used to rank chest X-rays in the same way that the AI model’s continuous probability score could be used. Therefore, an AUROC calculation would not be a fair representation of physician performance.

To compare the relative performance of AI and the four collaborative strategies, for each of the nine physicians, we calculated the performance metrics in a subset of images that the respective physician had reviewed. Unless otherwise stated, the average values and the 95% confidence intervals (CI) were generated by employing a bootstrapping approach with 1000 cluster bootstrap experiments. During each experiment, a sample set with replacement was drawn at the patient level to account for clustering of chest X-rays within patients, on which the performance metrics were calculated^25^ To estimate how each method (i.e., physicians, AI, and combinations of both) performs on average, the performance metrics were averaged across the nine physicians for each bootstrapping experiment leaving us with 1000 average values for each method. The 95% CI falls between the 2.5th and 97.5th percentiles of the bootstrap samples.

### Reporting summary

Further information on research design is available in the Nature Research Reporting Summary linked to this article.

## Supplementary information

supplementary material

REPORTING SUMMARY

## Data Availability

The datasets generated and/or analyzed during the current study were collected at Michigan Medicine. The University of Michigan’s Innovation Partnerships (UMIP) unit will handle potential charges/arrangements of the use of data by external entities, using such methods as material transfer agreements. Please contact UMIP (innovationpartnerships@umich.edu) for data inquiries.

## Code availability

A compiled version of the ARDS detection model can be made available to researchers from accredited research institutions after entering into a non-commercial license agreement with the University of Michigan. Please contact UMIP (innovationpartnerships@umich.edu) for inquiries. A related version of the algorithm is also available at ardsdetect.com. The code that produces the combinatory scores can be accessed at: github.com/weil-institute/physician-AI-collaboration-for-CXR-analysis.
