# Supplementary material for: Collaborative strategies for deploying artificial intelligence to complement physician diagnoses of acute respiratory distress syndrome
Source: NPJ Digit Med. 2023 Apr 8;6:62. doi: 10.1038/s41746-023-00797-9 (PMC10082784; doi:10.1038/s41746-023-00797-9)
Supplement: Supplementary file 1 — supplementary material [file 41746_2023_797_MOESM1_ESM.pdf]

Supplementary Information

Supplementary Tables

Supplementary Table 1. The accuracy of individual physicians in ARDS detection compared to the accuracy of other methods when applied to the same subset of images. The individual physicians for whom the physician-aided AI's performance was greater than the physician's performance are made bold. The individual physicians for whom the physician and physician-aided AI's performance was significantly different using the one-sided bootstrapped two-sample hypothesis testing are marked by asterisks. Numbers in parentheses indicate the 95% confidence intervals.

	**Physician**	**AI**	**AI-aided physician**	**Physician-aided AI**	**Average of physician & AI**	**Weighted average of physician & AI**
**Radiologist**	**0.839 (0.776, 0.899)**	0.842 (0.8, 0.888)	0.858 (0.797, 0.911)	**0.87 (0.822, 0.914)**	0.887 (0.834, 0.935)	0.879 (0.832, 0.923)
**Pulmonary attending 1**	**0.867 (0.816, 0.912)**	0.838 (0.798, 0.882)	0.867 (0.816, 0.912)	**0.882 (0.845, 0.918)**	0.867 (0.816, 0.912)	0.897 (0.86, 0.932)
**Pulmonary attending 2**	**0.747 (0.67, 0.82)**	0.84 (0.795, 0.884)	0.793 (0.731, 0.855)	**0.838 * (0.79, 0.886)**	0.834 (0.776, 0.886)	0.853 (0.803, 0.901)
**Pulmonary attending 3**	**0.85 (0.779, 0.918)**	0.832 (0.78, 0.885)	0.858 (0.791, 0.922)	**0.861 (0.811, 0.907)**	0.874 (0.818, 0.926)	0.868 (0.821, 0.913)
Pulmonary attending 4	0.852 (0.761, 0.923)	0.82 (0.758, 0.88)	0.847 (0.751, 0.92)	0.848 (0.773, 0.908)	0.861 (0.771, 0.93)	0.857 (0.776, 0.921)
**Pulmonary attending 5**	**0.598 (0.472, 0.721)**	0.803 (0.729, 0.871)	0.658 (0.542, 0.771)	**0.819 * (0.746, 0.889)**	0.774 (0.679, 0.866)	0.818 (0.746, 0.891)
**Pulmonary fellow 1**	**0.849 (0.779, 0.911)**	0.859 (0.801, 0.919)	0.859 (0.789, 0.921)	**0.889 (0.833, 0.942)**	0.885 (0.826, 0.937)	0.88 (0.824, 0.935)
**Pulmonary fellow 2**	**0.755 (0.624, 0.876)**	0.874 (0.82, 0.926)	0.761 (0.626, 0.879)	**0.859 * (0.795, 0.919)**	0.805 (0.699, 0.903)	0.837 (0.764, 0.906)
**Pulmonary fellow 3**	**0.911 (0.837, 0.974)**	0.912 (0.836, 0.974)	0.911 (0.837, 0.974)	**0.952 (0.908, 0.987)**	0.952 (0.902, 0.993)	0.952 (0.907, 0.988)

Supplementary Table 2. The sensitivity of individual physicians in ARDS detection compared to the sensitivity of other methods when applied to the same subset of images. The individual physicians for whom the physician-aided AI's performance was greater than the physician's performance are made bold. The individual physicians for whom the physician and physician-aided AI's performance was significantly different using the one-sided bootstrapped two-sample hypothesis testing are marked by asterisks. Numbers in parentheses indicate the 95% confidence intervals.

	**Physician**	**AI**	**AI-aided physician**	**Physician-aided AI**	**Average of physician & AI**	**Weighted average of physician & AI**
**Radiologist**	**0.388 (0.217, 0.566)**	0.744 (0.621, 0.858)	0.482 (0.3, 0.648)	**0.644 * (0.496, 0.778)**	0.586 (0.4, 0.745)	0.681 (0.528, 0.817)
**Pulmonary attending 1**	**0.684 (0.522, 0.817)**	0.746 (0.625, 0.857)	0.684 (0.522, 0.817)	**0.726 (0.596, 0.835)**	0.684 (0.522, 0.817)	0.737 (0.605, 0.848)
Pulmonary attending 2	0.937 (0.865, 1.0)	0.814 (0.692, 0.92)	0.952 (0.889, 1.0)	0.902 (0.827, 0.969)	0.94 (0.873, 0.99)	0.902 (0.817, 0.974)
**Pulmonary attending 3**	**0.856 (0.736, 0.946)**	0.797 (0.667, 0.905)	0.845 (0.727, 0.936)	**0.858 (0.779, 0.93)**	0.858 (0.756, 0.944)	0.846 (0.75, 0.932)
**Pulmonary attending 4**	**0.491 (0.219, 0.746)**	0.765 (0.587, 0.925)	0.491 (0.219, 0.746)	**0.666 * (0.432, 0.867)**	0.544 (0.292, 0.782)	0.614 (0.354, 0.833)
Pulmonary attending 5	0.955 (0.846, 1.0)	0.81 (0.6, 0.977)	0.931 (0.815, 1.0)	0.929 (0.833, 1.0)	0.953 (0.878, 1.0)	0.951 (0.871, 1.0)
Pulmonary fellow 1	0.787 (0.544, 0.947)	0.749 (0.596, 0.892)	0.787 (0.544, 0.947)	0.771 (0.586, 0.925)	0.792 (0.607, 0.944)	0.729 (0.543, 0.892)
Pulmonary fellow 2	0.91 (0.765, 1.0)	0.797 (0.656, 0.921)	0.909 (0.808, 1.0)	0.827 (0.697, 0.947)	0.857 (0.72, 0.974)	0.857 (0.72, 0.974)
**Pulmonary fellow 3**	**0.537 (0.4, 0.667)**	0.884 (0.737, 1.0)	0.537 (0.4, 0.667)	**0.84 * (0.714, 1.0)**	0.752 (0.611, 0.95)	0.84 (0.714, 1.0)

Supplementary Table 3. The specificity of individual physicians in ARDS detection compared to the specificity of other methods when applied to the same subset of images. The individual physicians for whom the physician-aided AI's performance was greater than the physician's performance are made bold. The individual physicians for whom the physician and physician-aided AI's performance was significantly different using the one-sided bootstrapped two-sample hypothesis testing are marked by asterrisks. Numbers in parentheses indicate the 95% confidence intervals.

	**Physician**	**AI**	**AI-aided physician**	**Physician-aided AI**	**Average of physician & AI**	**Weighted average of physician & AI**
Radiologist	0.994 (0.984, 1.0)	0.876 (0.827, 0.926)	0.987 (0.969, 1.0)	0.947 * (0.913, 0.976)	0.99 (0.978, 1.0)	0.947 (0.911, 0.978)
**Pulmonary attending 1**	**0.926 (0.871, 0.97)**	0.867 (0.816, 0.915)	0.926 (0.871, 0.97)	**0.932 (0.889, 0.967)**	0.926 (0.871, 0.97)	0.948 (0.913, 0.977)
**Pulmonary attending 2**	**0.7 (0.608, 0.787)**	0.846 (0.792, 0.896)	0.754 (0.676, 0.832)	**0.822 * (0.757, 0.88)**	0.807 (0.737, 0.875)	0.84 (0.776, 0.9)
**Pulmonary attending 3**	**0.848 (0.758, 0.931)**	0.841 (0.781, 0.898)	0.861 (0.781, 0.94)	**0.861 (0.801, 0.914)**	0.878 (0.81, 0.938)	0.874 (0.82, 0.925)
Pulmonary attending 4	0.987 (0.967, 1.0)	0.84 (0.766, 0.904)	0.981 (0.956, 1.0)	0.916 * (0.851, 0.965)	0.981 (0.956, 1.0)	0.949 (0.912, 0.979)
**Pulmonary attending 5**	**0.504 (0.373, 0.648)**	0.799 (0.715, 0.871)	0.586 (0.459, 0.718)	**0.79 * (0.701, 0.876)**	0.726 (0.61, 0.842)	0.783 (0.691, 0.87)
**Pulmonary fellow 1**	**0.867 (0.785, 0.935)**	0.892 (0.817, 0.959)	0.88 (0.799, 0.944)	**0.924 * (0.869, 0.973)**	0.912 (0.85, 0.963)	0.924 (0.867, 0.979)
**Pulmonary fellow 2**	**0.719 (0.572, 0.858)**	0.891 (0.83, 0.951)	0.726 (0.579, 0.862)	**0.866 * (0.793, 0.94)**	0.793 (0.667, 0.908)	0.832 (0.75, 0.916)
Pulmonary fellow 3	1.0 (1.0, 1.0)	0.92 (0.83, 0.989)	1.0 (1.0, 1.0)	0.979 * (0.946, 1.0)	1.0 (1.0, 1.0)	0.979 (0.947, 1.0)

Supplementary Figures

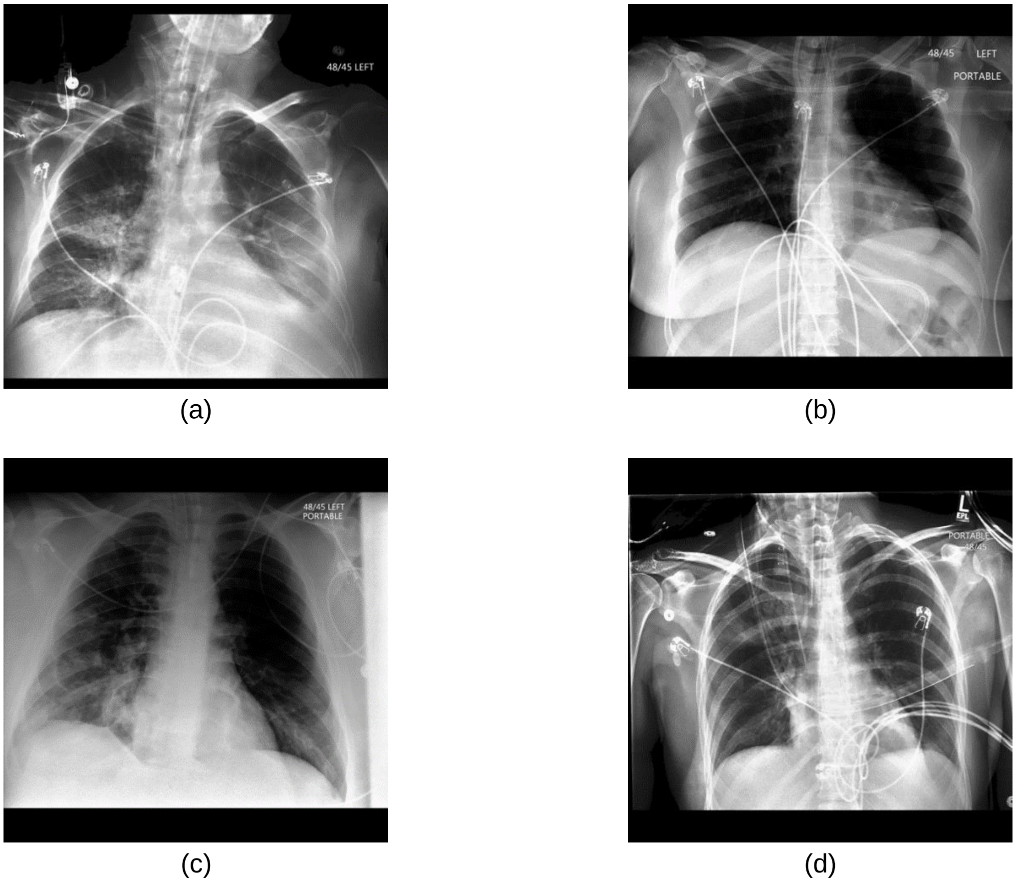

Supplementary Figure 1. (a) a chest X-ray consistent with ARDS that is correctly classified as ARDS positive by the AI model, (b) a chest X-ray not consistent with ARDS that is correctly classified as ARDS negative by the AI model, (c) a chest X-ray consistent with ARDS that is incorrectly classified as ARDS negative by the AI model, (d) a chest X-ray not consistent with ARDS that is incorrectly classified as ARDS positive by the AI model.

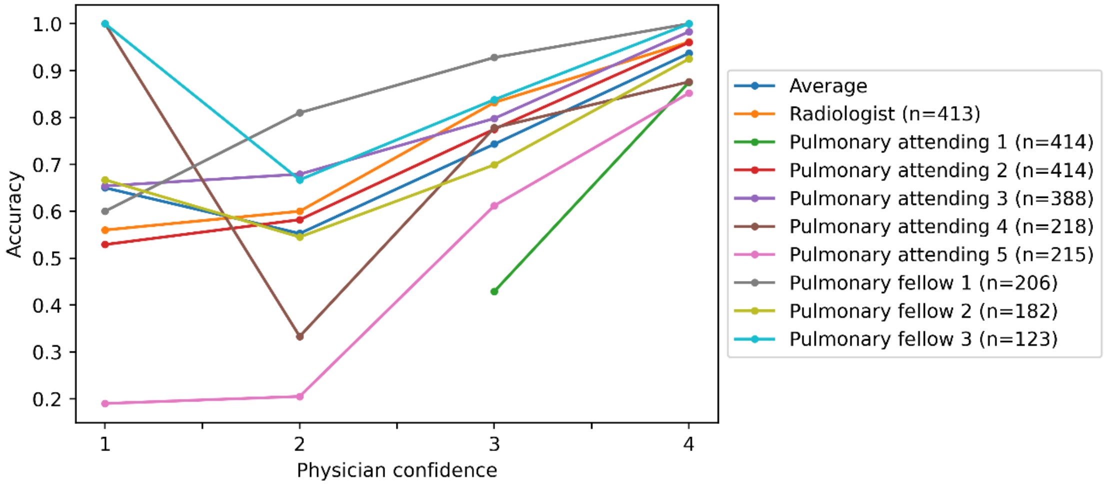

Supplementary Figure 2. Physicians' accuracies stratified on their respective confidence. Attending physician 1 specified either high or moderate confidence in their decision (confidence = 3 or 4) thus there is no sample with the confidence of 1 or 2 to report the physician's accuracy on. The numbers in parentheses specify the total number of chest X-rays that a physician reviewed. Bootstrapping was not used to generate Supplementary Figure 2.

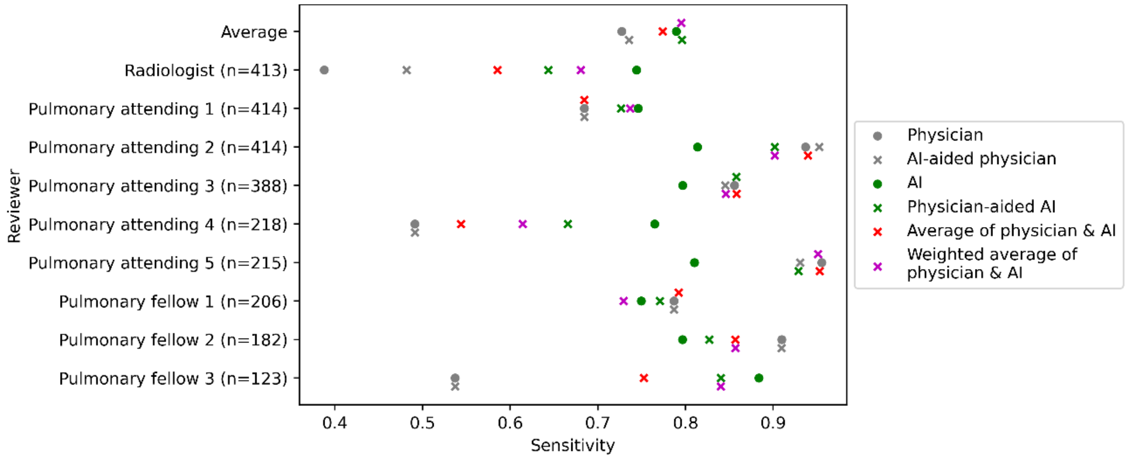

(a)

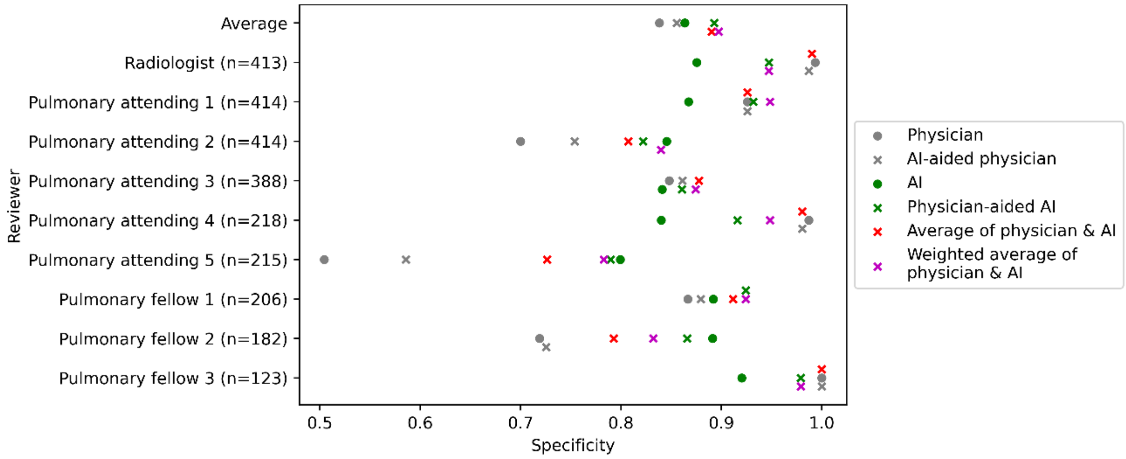

(b)

Supplementary Figure 3. (a), and (b), respectively, show the sensitivity, and specificity of individual physicians, along with the performance of AI and four combinatory strategies when assessed on the same subset as physicians. Numbers in parentheses indicate the number of chest X-rays that each physician read.

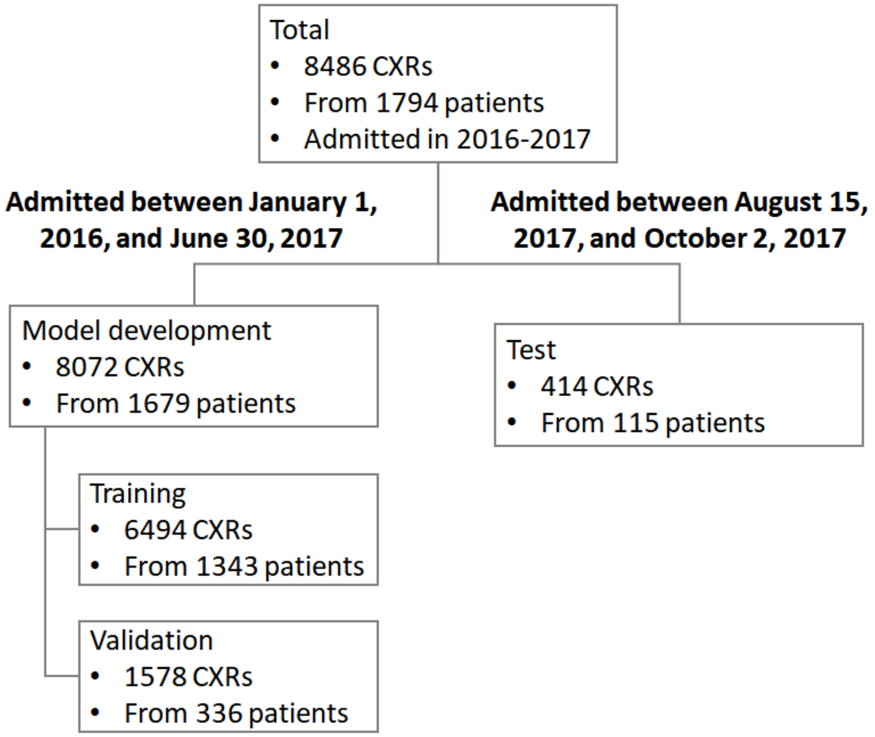

Supplementary Figure 4. Flow diagram of patients. Patients who were admitted to the University of Michigan hospital in 2016-2017 with acute hypoxic respiratory failure were included in the study. Chest X-rays performed in the first 7 days after-admission were analyzed. Patients admitted between Jan 1, 2016 and June 30, 2017 were included in the development set, with patients further split randomly into the training and validation sets. Patients in the test set were admitted between August 15 and October 2, 2017. Patients in training, validation, and test sets are mutually exclusive. *CXR* chest X-ray.

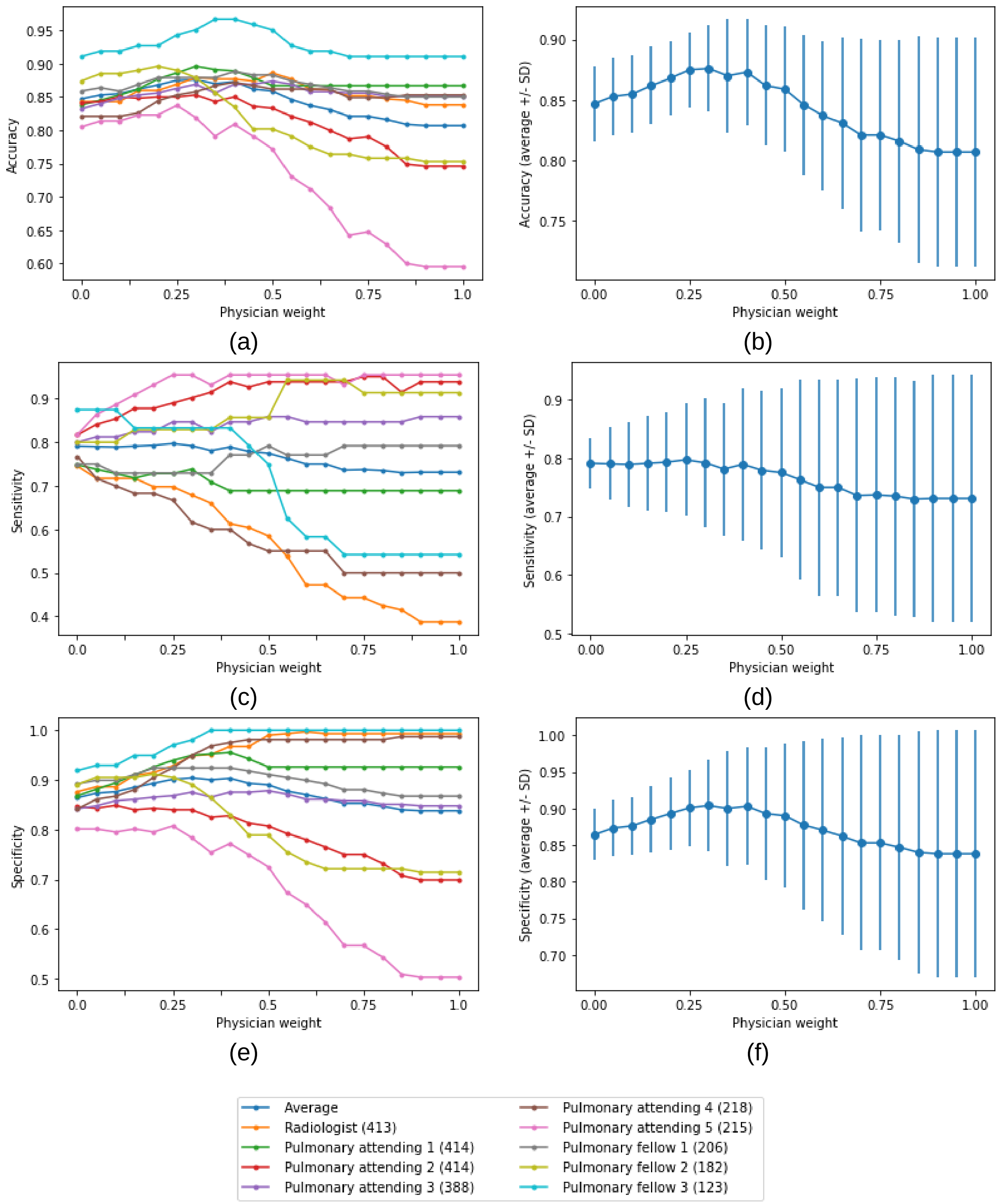

Supplementary Figure 5. (a), (c), and (e), respectively, show each physician's accuracy, sensitivity, and specificity with respect to their weight while using the weighted averaging method. AI's weight is equal to 1 - physician weight. (b), (d), and (f), respectively, show the average physician's accuracy ± standard deviation (SD), sensitivity ± SD, and specificity ± SD. The numbers in parentheses specify the total number of chest X-rays that a physician reviewed. Bootstrapping was not used to generate Supplementary Figure 5.
