# Supplementary material for: Collaborative strategies for deploying artificial intelligence to complement physician diagnoses of acute respiratory distress syndrome
Source: NPJ Digit Med. 2023 Apr 8;6:62. doi: 10.1038/s41746-023-00797-9 (PMC10082784; doi:10.1038/s41746-023-00797-9)
Supplement: Supplementary file 2 — REPORTING SUMMARY [file 41746_2023_797_MOESM2_ESM.pdf]

nature portfolio

Corresponding author(s): DBPR 04885

Last updated by author(s): Jan 17, 2023

Reporting Summary

Nature Portfolio wishes to improve the reproducibility of the work that we publish. This form provides structure for consistency and transparency in reporting. For further information on Nature Portfolio policies, see our Editorial Policies and the Editorial Policy Checklist.

Statistics

For all statistical analyses, confirm that the following items are present in the figure legend, table legend, main text, or Methods section.

n/a	Confirmed	
☐	☒	The exact sample size (*n*) for each experimental group/condition, given as a discrete number and unit of measurement
☐	☒	A statement on whether measurements were taken from distinct samples or whether the same sample was measured repeatedly
☐	☒	The statistical test(s) used AND whether they are one- or two-sided<br>*Only common tests should be described solely by name; describe more complex techniques in the Methods section.*
☐	☒	A description of all covariates tested
☒	☐	A description of any assumptions or corrections, such as tests of normality and adjustment for multiple comparisons
☐	☒	A full description of the statistical parameters including central tendency (e.g. means) or other basic estimates (e.g. regression coefficient) AND variation (e.g. standard deviation) or associated estimates of uncertainty (e.g. confidence intervals)
☐	☒	For null hypothesis testing, the test statistic (e.g. *F*, *t*, *r*) with confidence intervals, effect sizes, degrees of freedom and *P* value noted<br>*Give P values as exact values whenever suitable.*
☒	☐	For Bayesian analysis, information on the choice of priors and Markov chain Monte Carlo settings
☒	☐	For hierarchical and complex designs, identification of the appropriate level for tests and full reporting of outcomes
☒	☐	Estimates of effect sizes (e.g. Cohen's *d*, Pearson's *r*), indicating how they were calculated

Our web collection on statistics for biologists contains articles on many of the points above.

Software and code

Policy information about availability of computer code

Data collection	The 414 chest X-ray included in the test set were from 115 patients consecutively hospitalized between August 15 to October 2, 2017 at the University of Michigan who met criteria for acute hypoxemic respiratory failure (AHRF) in one of 4 intensive care units (medical, surgical, cardiac, and trauma). The University of Michigan Institutional Review Board (IRB) approved this study (HUM00180748) with a waiver of informed consent from patient subjects and study physicians.
Data analysis	Python programming language was used to perform all modeling and analyses.

For manuscripts utilizing custom algorithms or software that are central to the research but not yet described in published literature, software must be made available to editors and reviewers. We strongly encourage code deposition in a community repository (e.g. GitHub). See the Nature Portfolio guidelines for submitting code & software for further information.

Data

Policy information about availability of data

All manuscripts must include a data availability statement. This statement should provide the following information, where applicable:

- Accession codes, unique identifiers, or web links for publicly available datasets
- A description of any restrictions on data availability
- For clinical datasets or third party data, please ensure that the statement adheres to our policy

The dataset used in the current study were collected at Michigan Medicine. The University of Michigan's Innovation Partnerships (UMIP) unit will handle potential

charges/arrangements of the use of data by external entities, using such methods as material transfer agreements. Please contact UMIP (innovationpartnerships@umich.edu) for data inquiries.

Human research participants

Policy information about studies involving human research participants and Sex and Gender in Research.

Reporting on sex and gender	The test dataset included 414 chest X-rays from 115 adult patients, 46 female and and 69 male. Due to the limited number of ARDS chest X-rays across patient demographic subgroups, we could not provide conclusive evidence for either accepting or rejecting the generalizability of the collaborative strategies across patients subgroups. Thus sex-based analyses have not been performed.
Population characteristics	The median [Q1, Q3] age of patients who developed ARDS are 59 [49.25, 67], while these numbers are 63 [55, 72] for patients without ARDS. There is no significant different between the age distribution of two groups. We also found no significant difference between body mass index (BMI) and Race of patients with and without ARDS.
Recruitment	Patient informed consent was not required given that this was a retrospective investigation. The 414 chest X-ray included in the test set were from 115 patients consecutively hospitalized between August 15 to October 2, 2017 at the University of Michigan who met criteria for acute hypoxemic respiratory failure (AHRF) in one of 4 intensive care units (medical, surgical, cardiac, and trauma)
Ethics oversight	The University of Michigan Institutional Review Board (IRB) approved this study (HUM00180748).

Note that full information on the approval of the study protocol must also be provided in the manuscript.

Field-specific reporting

Please select the one below that is the best fit for your research. If you are not sure, read the appropriate sections before making your selection.

☒ Life sciences ☐ Behavioural & social sciences ☐ Ecological, evolutionary & environmental sciences

For a reference copy of the document with all sections, see nature.com/documents/nr-reporting-summary-flat.pdf

Life sciences study design

All studies must disclose on these points even when the disclosure is negative.

Sample size	The 414 chest X-ray included in the test set were from 115 patients consecutively hospitalized between August 15 to October 2, 2017 at the University of Michigan who met criteria for acute hypoxemic respiratory failure (AHRF) in one of 4 intensive care units (medical, surgical, cardiac, and trauma).
Data exclusions	Patients who did not meet the criteria for acute hypoxemic respiratory failure (AHRF) are excluded. AHRF was defined as patients who had a $PaO_2/FiO_2 < 300$ while receiving invasive mechanical ventilation or non-invasive mechanical ventilation.
Replication	NA
Randomization	NA
Blinding	Physicians who labeled the chest X-rays also reviewed other relevant clinical information for each patient.

Reporting for specific materials, systems and methods

We require information from authors about some types of materials, experimental systems and methods used in many studies. Here, indicate whether each material, system or method listed is relevant to your study. If you are not sure if a list item applies to your research, read the appropriate section before selecting a response.

Materials & experimental systems

n/a	Involved in the study	
☒	☐	Antibodies
☒	☐	Eukaryotic cell lines
☒	☐	Palaeontology and archaeology
☒	☐	Animals and other organisms
☐	☒	Clinical data
☒	☐	Dual use research of concern

Methods

n/a	Involved in the study	
☒	☐	ChIP-seq
☒	☐	Flow cytometry
☒	☐	MRI-based neuroimaging

Clinical data

Policy information about clinical studies

All manuscripts should comply with the ICMJE guidelines for publication of clinical research and a completed CONSORT checklist must be included with all submissions.

Clinical trial registration: NA

Study protocol: NA

Data collection: This is a secondary analysis of 115 patients consecutively hospitalized between August 15 to October 2, 2017 at the University of Michigan who met criteria for acute hypoxemic respiratory failure (AHRF) in one of 4 intensive care units (medical, surgical, cardiac, and trauma).

Outcomes: The findings of acute respiratory distress syndrome (ARDS) on chest X-rays are used as an outcome.
